# Supplementary material for: Mutational profiling of SARS-CoV-2 papain-like protease reveals requirements for function, structure, and drug escape
Source: Nat Commun. 2024 Jul 23;15:6219. doi: 10.1038/s41467-024-50566-9 (PMC11266423; doi:10.1038/s41467-024-50566-9)
Supplement: Supplementary file 1 — Supplementary Information [file 41467_2024_50566_MOESM1_ESM.pdf]

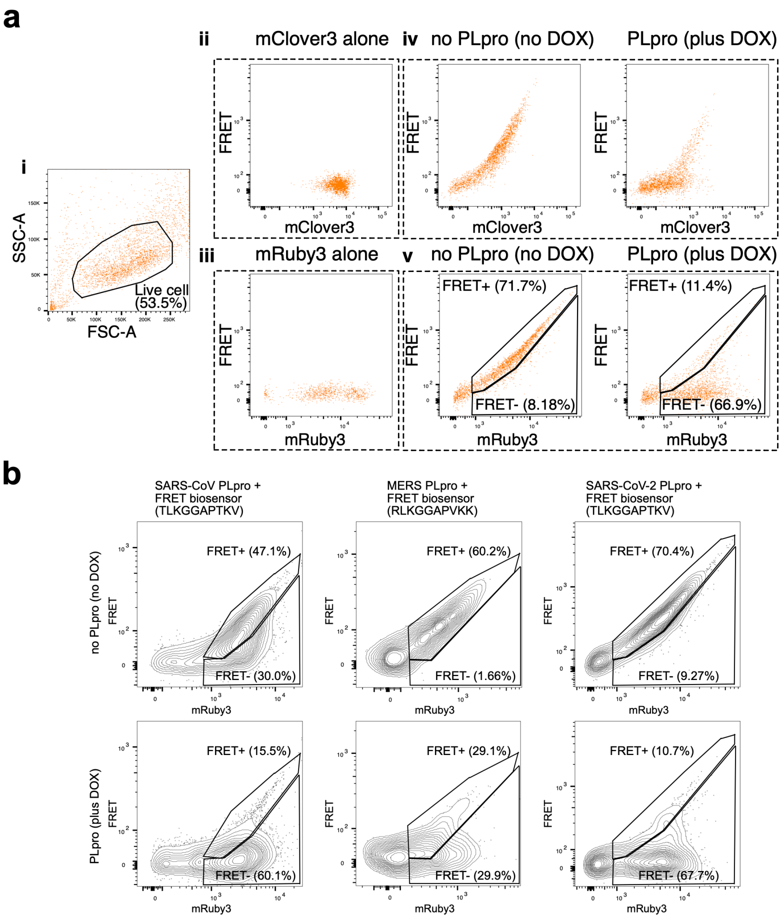

Supplementary Figure 1. **PLpro FRET assay and other coronavirus biosensors**. **a)** Characterization of the SARS-CoV-2 PLpro biosensor. i) FSC/SSC gating of HEK293T cells. ii) Flow cytometry plot of 293T cells expressing mClover3 (donor). iii) Flow cytometry plot of 293T cells expressing mRuby3 (acceptor). No signal is seen in the FRET channel when donor and acceptor are expressed independently. iv) 293T biosensor cells in the absence (left) and presence (right) of PLpro with FRET on the y-axis and mClover3 on the x-axis. Fluorescence in the donor channel unexpectedly dropped upon biosensor cleavage. The reduction of mClover3 fluorescence indicates that free mClover3 fused to a fragment of the PLpro cleavage motif ($T_{P5}L_{P4}K_{P3}G_{P2}G_{P1}$) is less stable than the intact biosensor. We therefore present data as FRET versus acceptor fluorescence. v) shows the same cells as iv), with mRuby3 on the x-axis. Biosensor cleavage reduced FRET and resulted in unexpected drop in mClover3 fluorescence so gating for analysis and selection was performed on mRuby3 vs FRET flow cytometry plots. **b)** Flow cytometry contour plots illustrating the sensitivity of biosensors to SARS-CoV (left), MERS (middle) and SARS-CoV-2 (right) PLpro. Upper row shows biosensor fluorescence in the absence of PLpro, lower row in the presence.

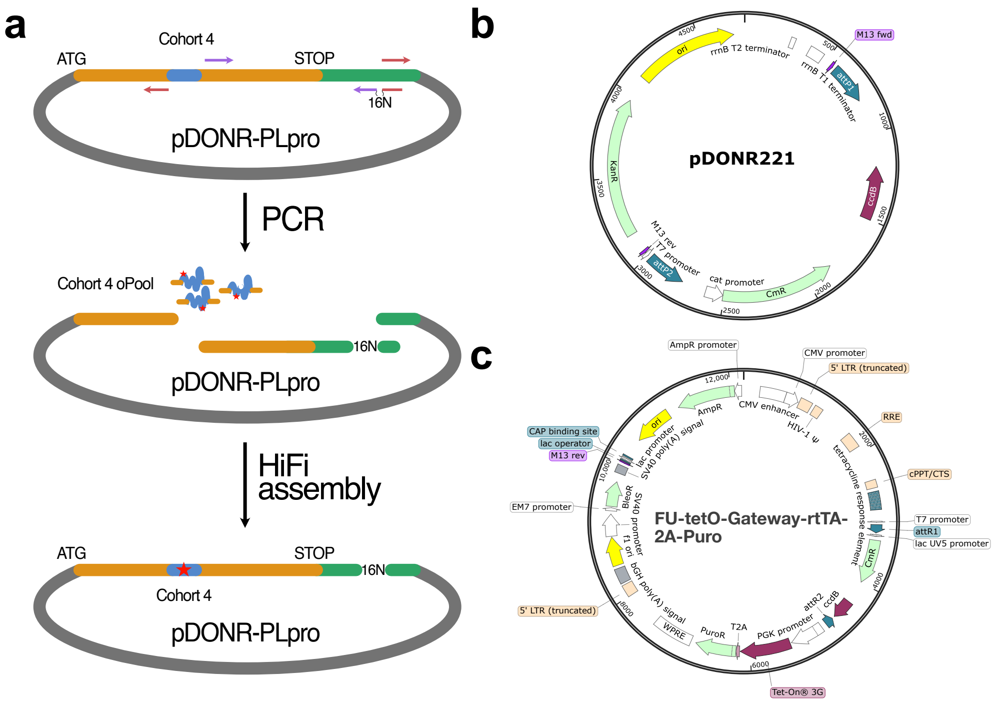

Supplementary Figure 2. **Mutagenesis strategy and vector maps. a)** Mutagenesis strategy of PLpro DMS library, cohort 4 showing primer binding sites to generate the vector fragment and 3' barcoded PLpro fragment with PCR. These two fragments were mixed with cohort 4 IDT oPools containing degenerate codons and assembled with NEB HiFi assembly. **b)** Vector map of pDONR221 **c)** Vector map of FU-tetO-Gateway-rtTA-2A-Puro. DNA sequences are provided in source data.

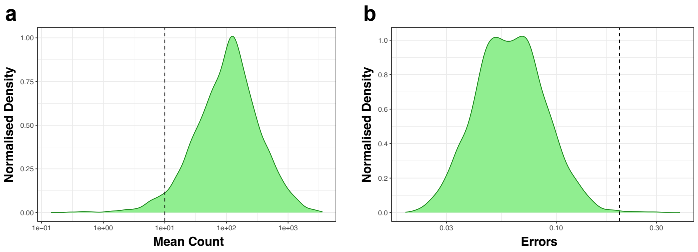

Supplementary Figure 3. **Activity Data filters**. **a)** The average reads per variant in the activity dataset depicted as a density distribution plot. Reads with less than or equal to 10 mean counts were filtered. **b)** The distribution of errors for variants in the activity dataset. Variants with errors less than 0.2 were kept. Source data are provided as a Source Data file.

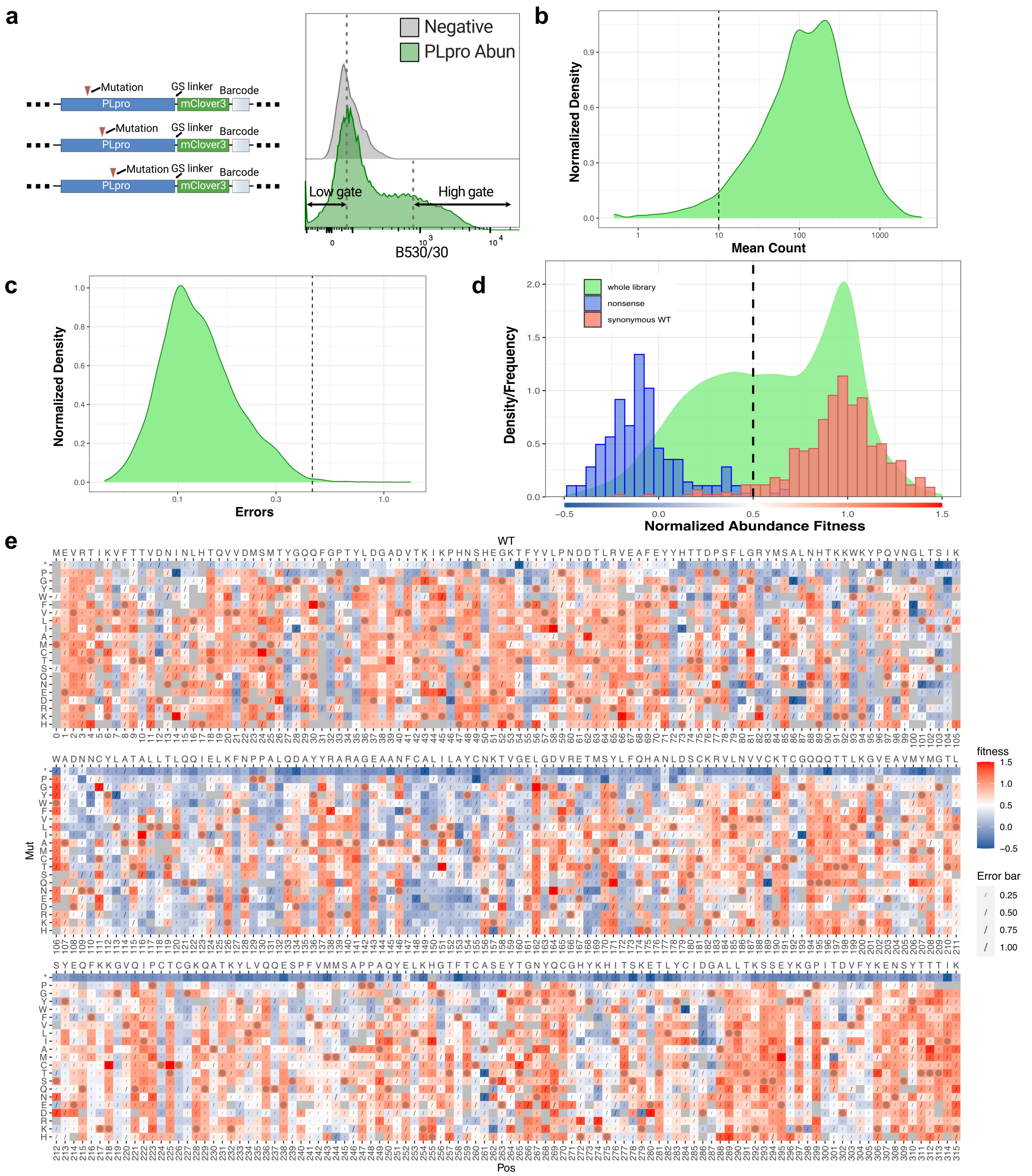

Supplementary Figure 4. **Abundance assay design and Sequence-Function map. a)** Cartoon of the PLpro-mClover3 fusion library (*left*) and flow cytometry plot (*right*) of mClover3 fluorescence in 293T cells before (grey) and after (green) introduction of PLpro abundance library. Cells were sorted according to the high and low gates marked. **b)** The average reads per variant in the abundance dataset depicted as a density distribution plot. Reads with less than or equal to 10 mean counts were filtered. **c)** The distribution of errors for variants in the abundance dataset. Variants with errors less than 0.45 were kept. **d)** The distribution of normalized abundance dataset fitness scores for the whole library (density; green), overlaid with the frequency of scores from synonymous wildtype variants (red; set at 1) and nonsense variants at positions 1-305 (blue; set at 0). The color bar below the x-axis matches the color scheme used in e). **e)** The normalized sequence-function map of abundance data from 2 independent transductions (see *supplementary file* **2** for replicate scores). The bottom x-axis indicates the position; the top x-axis indicates WT sequence; and the y-axis indicates the mutation. Fitness is shown in a two-color gradient with red indicating abundant variants, blue low abundant variants, and grey missing variants. The white point of the gradient was set at 0.5. Wildtype variants are marked with a circle. Errors are indicated with a diagonal line or if above 1, an X. Source data are provided as a Source Data file.

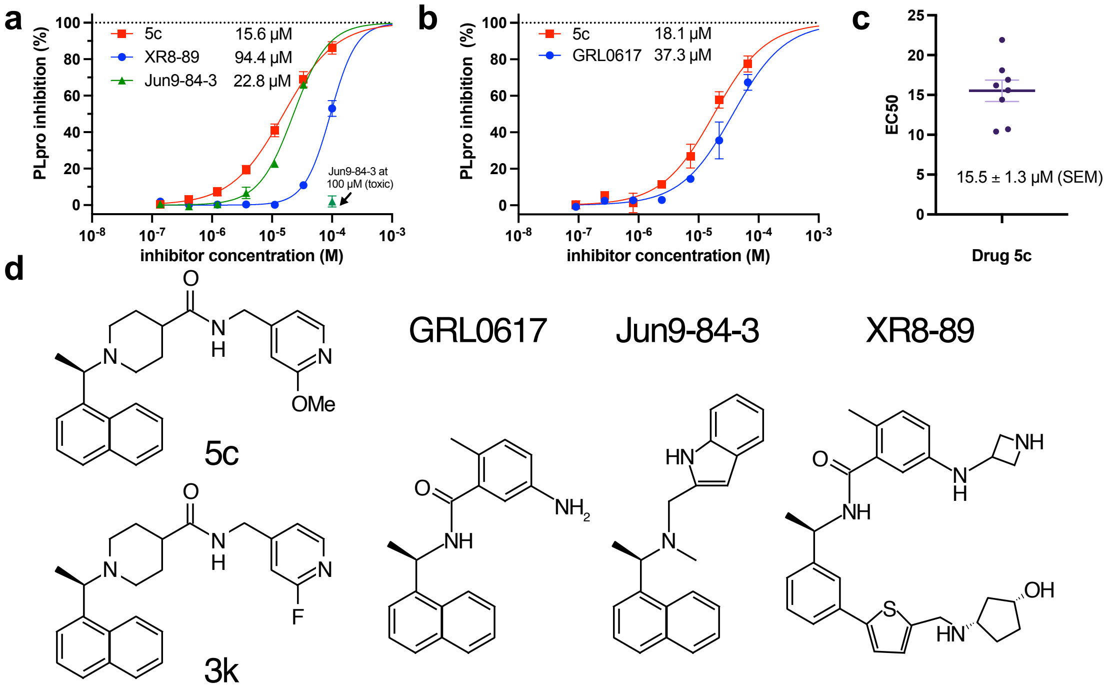

Supplementary Figure 5. **Dose response of lead PLpro compounds in the cellular FRET assay**. **a)** Dose response of 5c, Jun9-84-3 and XR8-89 (technical triplicates). Error bars: mean ± SD **b)** Dose response of 5c and GRL0617 (technical triplicates). Error bar: mean ± SD; Data was normalized so the top was 100 and bottom was 0 after fitting the 5c dose response curve that had a hillslope of 1. XR8-89 and Jun9-84-3 had hillslopes of 2 and 1.5 respectively. EC50 values for each experiment are marked, N = 1. **c)** Plot C shows the EC50s of eight independent measurements of 5c, which had a mean of 15.5 µM, and standard error of the mean (SEM) of 1.3 µM. **d)** Chemical structures of compounds referred to in this study. Source data are provided as a Source Data file.

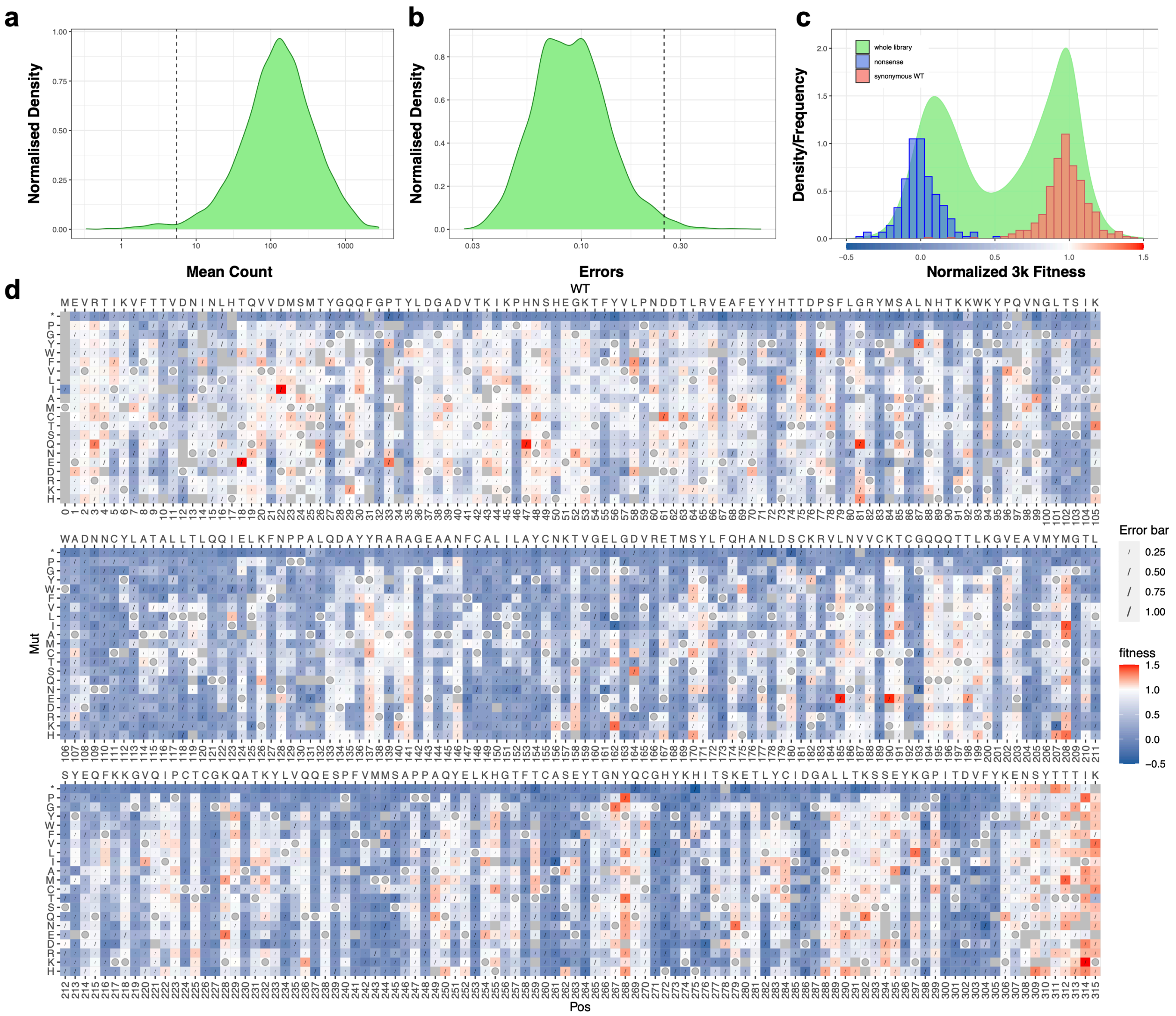

Supplementary Figure 6. **Sequence-function map of normalized 3k drug-escape fitness scores**. **a)** The average reads per variant in the 3k drug-escape dataset depicted as a density distribution plot. Reads with less than or equal to 5.5 mean counts were filtered. **b)** The distribution of errors for variants in the 3k drug-escape dataset. Variants with errors less than 0.25 were kept. **c)** The distribution of normalized 3k drug-escape fitness scores for the whole library (density; green), overlaid with the frequency of scores from synonymous wildtype variants (red; set at 1) and nonsense variants at positions 1-305 (blue; set at 0). The color bar below the x-axis matches the color scheme used in d). **d)** The normalized sequence-function map of the data from 3 independent transductions. The bottom x-axis indicates the position; the top x-axis indicates WT sequence; and the y-axis indicates the mutation. Fitness is shown in a three-color gradient with red indicating escape variants, blue non-escape variants, and the white point set at the wild-type score of 1. Grey indicates missing variants. Wildtype variants are marked with a circle. Errors are indicated with a line. Source data are provided as a Source Data file.

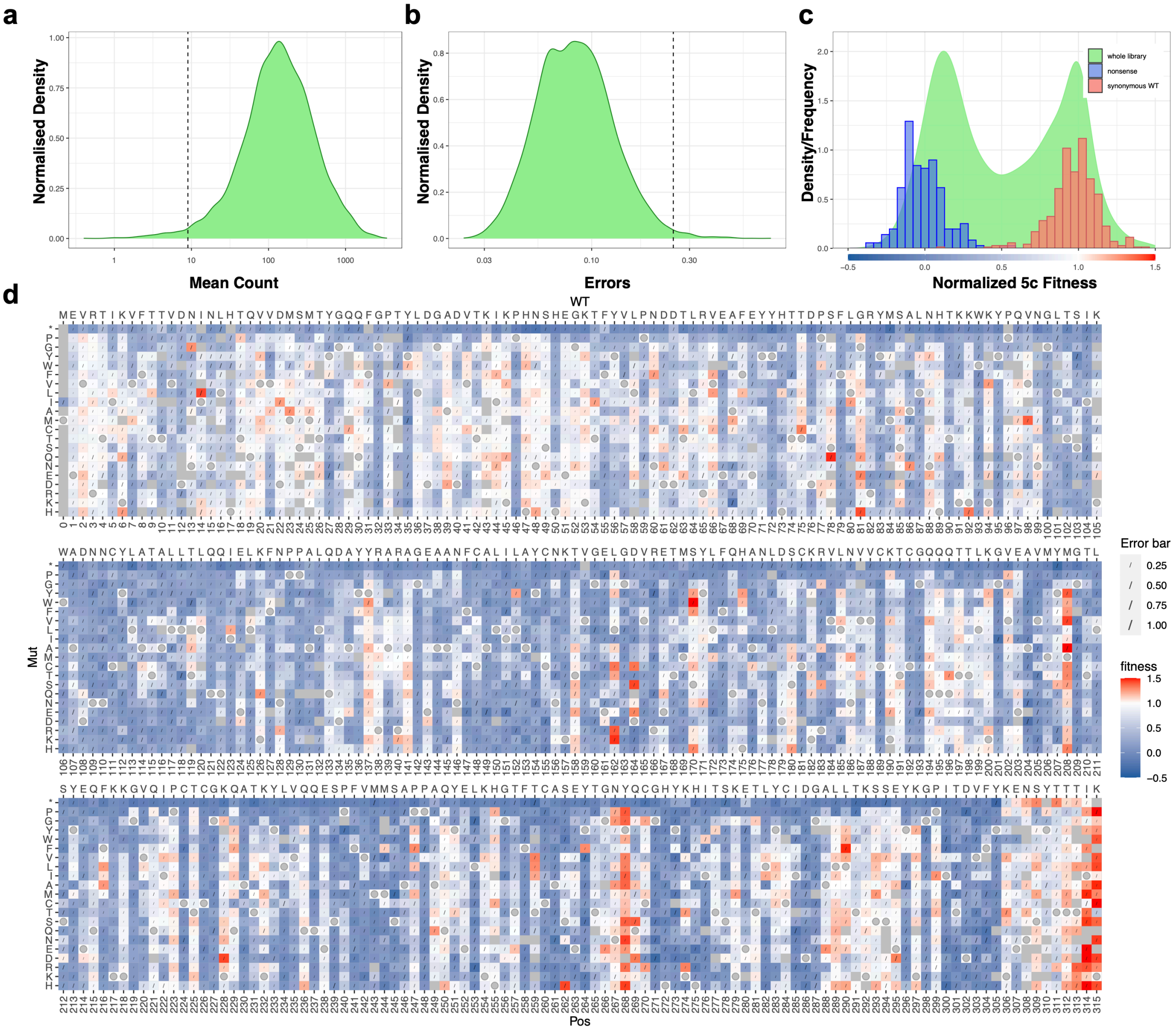

Supplementary Figure 7. **Sequence-function map of normalized 5c drug-escape fitness scores**. **a)** The average reads per variant in the 5c drug-escape dataset depicted as a density distribution plot. Reads with less than or equal to 9 mean counts were filtered. **b)** The distribution of errors for variants in the 5c drug-escape dataset. Variants with errors less than 0.25 were kept. **c)** The distribution of normalized 5c drug-escape fitness scores for the whole library (density; green), overlaid with the frequency of scores from synonymous wildtype variants (red; set at 1) and nonsense variants at positions 1-305 (blue; set at 0). The color bar below the x-axis matches the color scheme used in d). **d)** The normalized sequence-function map of the data from 5 independent transductions. The bottom x-axis indicates the position; the top x-axis indicates WT sequence; and the y-axis indicates the mutation. Fitness is shown in a three-color gradient with red indicating escape variants, blue non-escape variants, and the white point set at the wild-type score of 1. Grey indicates missing variants. Wildtype variants are marked with a circle. Errors (sigma) are indicated with a line. Source data are provided as a Source Data file.

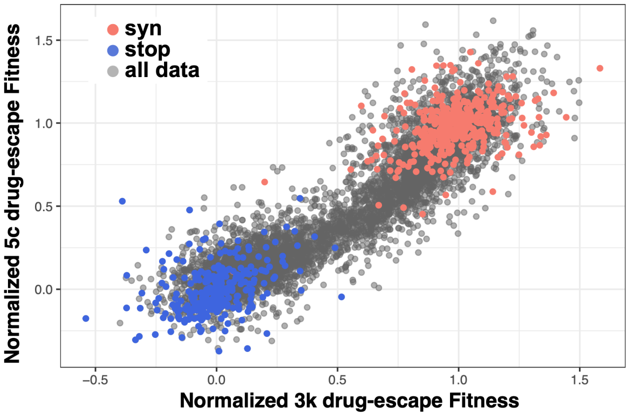

Supplementary Figure 8. **Correlation between normalized drug-escape scores between 3k and 5c and structures of both compounds***.* Plot of Normalized 3k (x-axis) and 5c (y-axis) drug-escape fitness scores. Nonsense variants (positions 1-305) are shown in blue, synonymous WT are shown in red and the rest shown in grey. Source data are provided as a Source Data file.

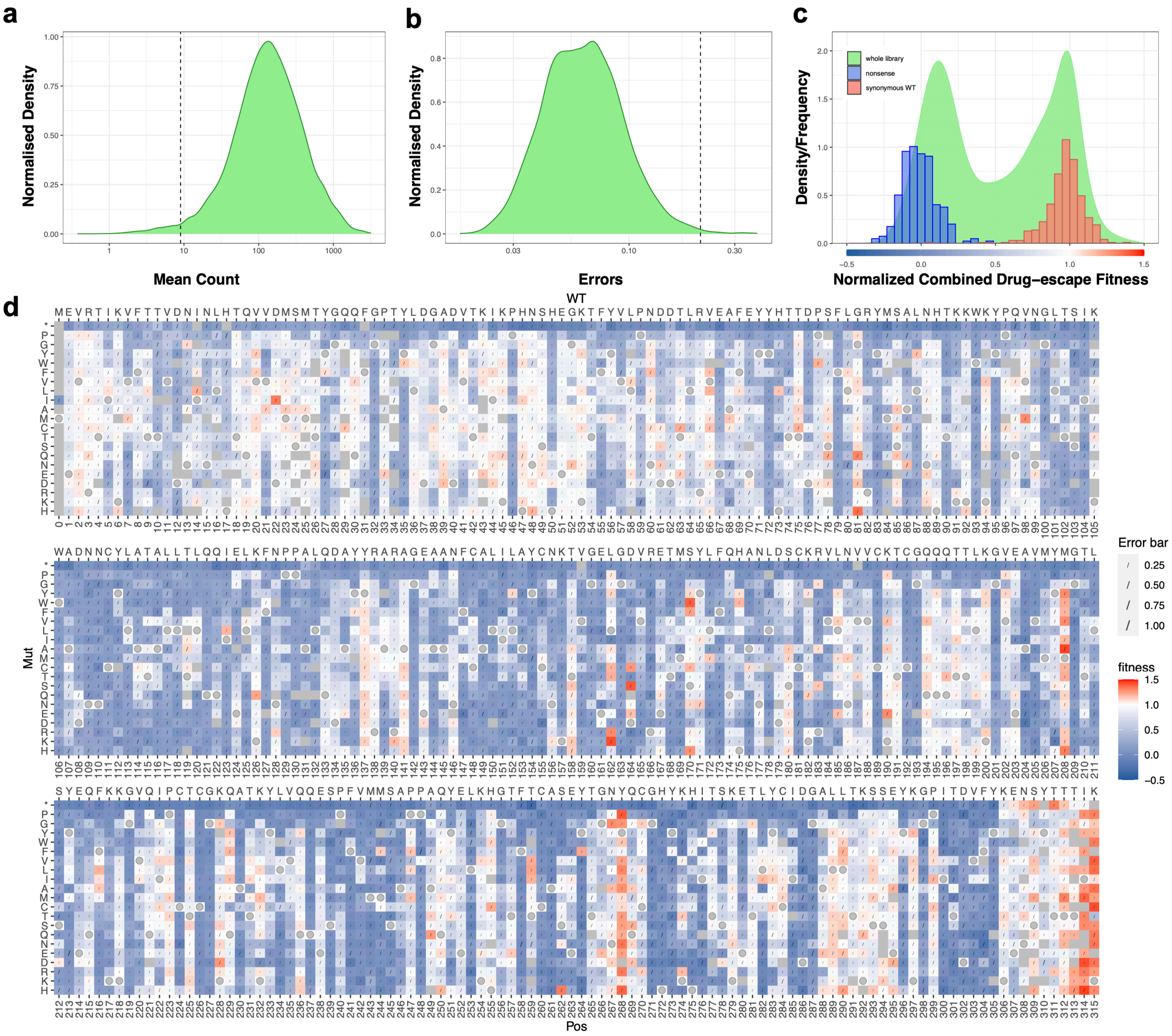

Supplementary Figure 9. **Sequence-function map of normalized 3k & 5c combined drug-escape fitness scores**. **a)** The average reads per variant in the combined drug-escape dataset depicted as a density distribution plot. Reads with less than or equal to 9 mean counts were filtered. **b)** The distribution of errors for variants in the combined drug-escape dataset. Variants with errors less than 0.21 were kept. **c)** The distribution of normalized combined drug-escape fitness scores for the whole library (density; green), overlaid with the frequency of scores from synonymous wildtype variants (red; set at 1) and nonsense variants at positions 1-305 (blue; set at 0). The color bar below the x-axis matches the color scheme used in d). **d)** The normalized sequence-function map of the data. The bottom x-axis indicates the position; the top x-axis indicates WT sequence; and the y-axis indicates the mutation. Fitness is shown in a three-color gradient with red indicating escape variants, blue non-escape variants, and the white point set at the wild-type score of 1. Grey indicates missing variants. Wildtype variants are marked with a circle. Errors (sigma) are indicated with a line. Source data are provided as a Source Data file.

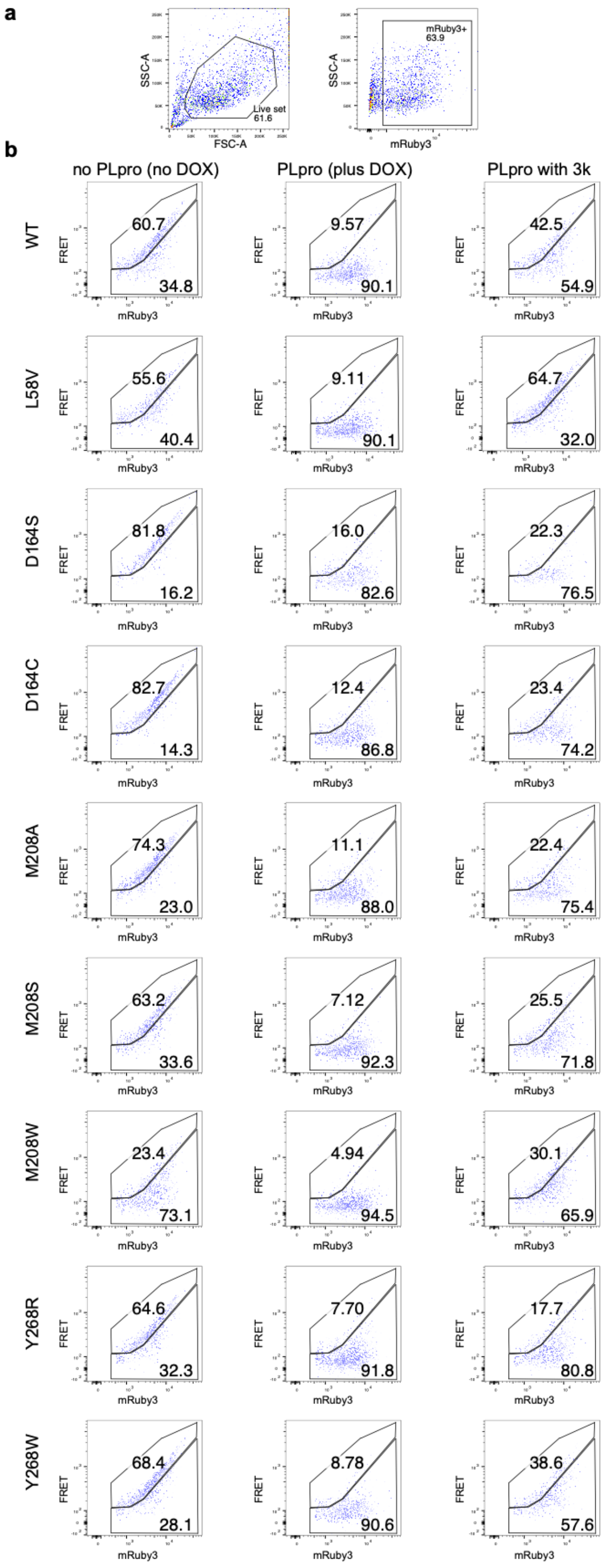

Supplementary Figure 10. **Flow cytometry plots of PLpro variants.** a) Gating Strategy for plots in b). FSC and SSC was used to select live cells, followed by mRuby3 and SSC to select cells containing the biosensor. b) Each row represents the PLpro variant indicated; Conditions are shown by columns. The first column contains cells with no dox treatment; the second column contains cells treated with DOX; the third column contains cells treated with DOX and 3k. The x-axis is mRuby3 (YG610/20) and y-axis is FRET (B610/20). In each plot, the upper gate shows FRET positive cells (cells with no PLpro activity) and the lower gate FRET negative (cells with PLpro activity). Percentages of cells in each gate are shown. One independent transduction was tested twice with similar results.

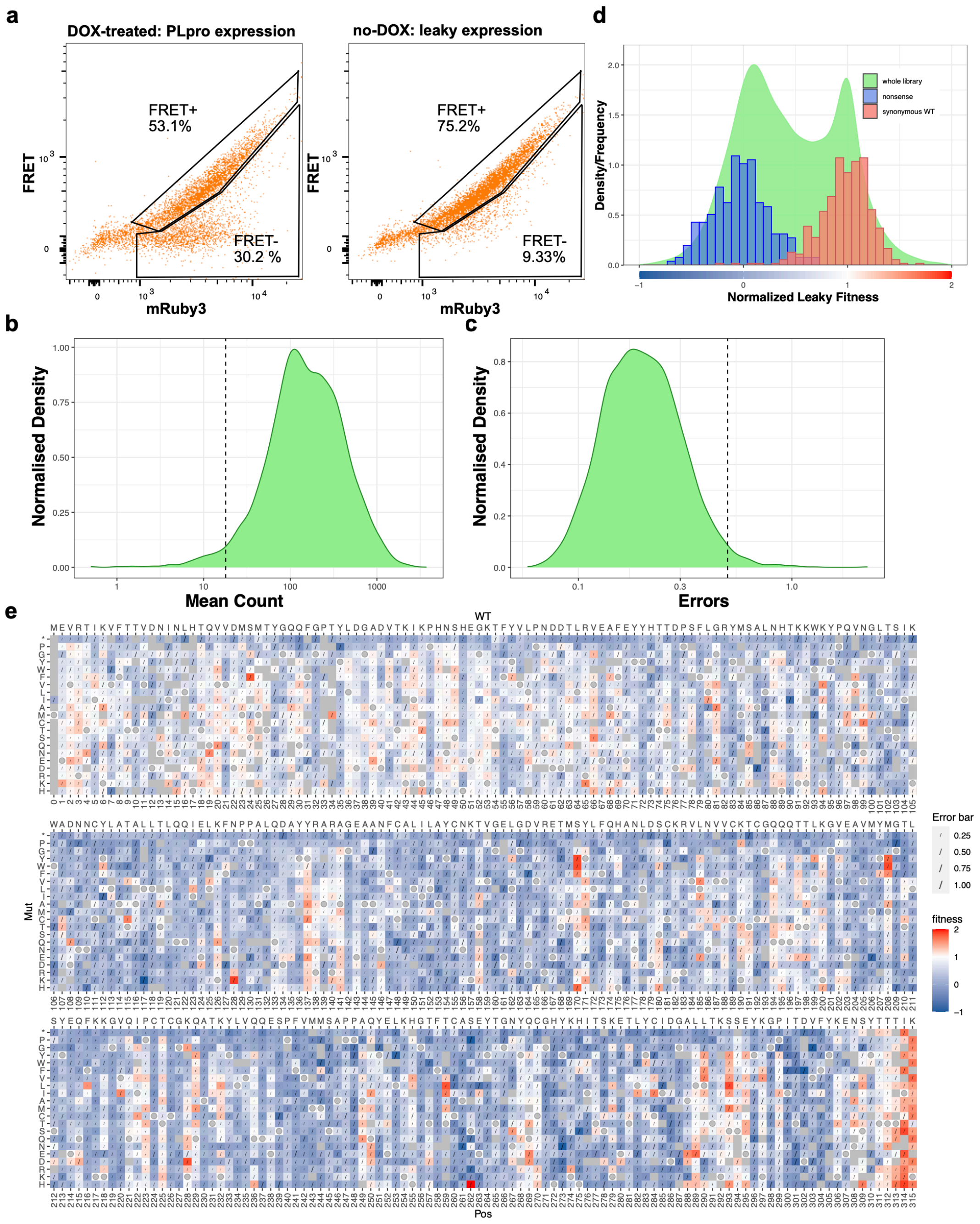

Supplementary Figure 11. **Normalized PLpro leaky expression sequence-function map**. **a)** Flow cytometry plots of the PLpro activity library in the presence of dox (left) and in the absence (right). The x-axis is mRuby3 (YG610/20) and y-axis is FRET (B610/20). **b)** The average reads per variant in the leaky expression dataset depicted as a density distribution plot. Reads with less than or equal to 18 mean counts were filtered. **c)** The distribution of errors for variants in the leaky expression dataset. Variants with errors less than 0.5 were kept. **d)** The distribution of normalized leaky expression fitness scores for the whole library (density; green), overlaid with the frequency of scores from synonymous wildtype variants (red; set at 1) and nonsense variants at positions 1-305 (blue; set at 0). The color bar below the x-axis matches the color scheme used in e). **e)** The normalized sequence-function map of the data from 2 independent transductions. The bottom x-axis indicates the position; the top x-axis indicates WT sequence; and the y-axis indicates the mutation. Fitness is shown in a three-color gradient with red indicating active and leaky variants, blue not leaky or inactive variants, and the white point set at the wild-type score of 1. Grey indicates missing variants. Wildtype variants are marked with a circle. Errors (sigma) are indicated with a line. Source data are provided as a Source Data file.

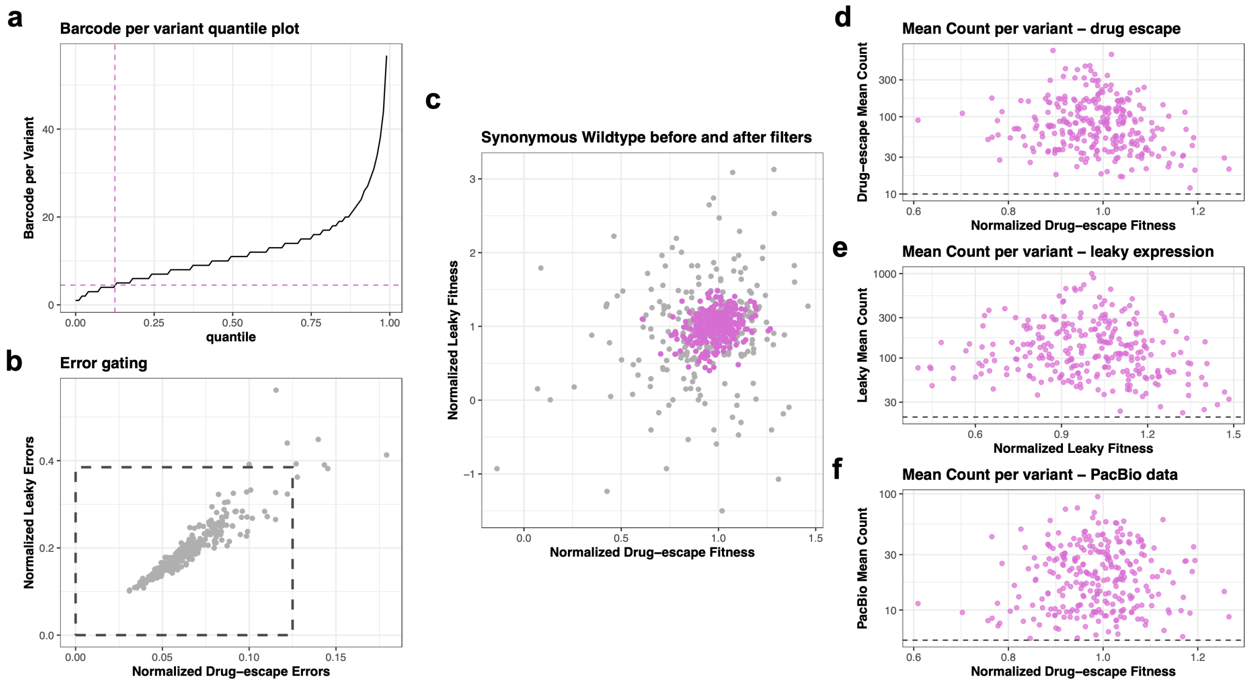

Supplementary Figure 12. **Noise reduction to allow comparison between combined drug-escape and leaky expression fitness scores guided by synonymous wildtype fitness scores**. **a)** Barcodes per variant quantile plot. Based on this plot, variants with less than 5 barcodes were filtered removing ~12% of the data. **b)** Plot of errors seen in drug-escape and leaky-expression fitness scores. Variants with high errors in both datasets were removed. The dashed rectangle indicates data that passed selection. **c)** The distribution of synonymous wildtype drug-escape and leaky-expression fitness scores after filters from (a) and (b) were applied. Data before filtering is shown in grey and after filtering is shown in magenta. **d-e)** The synonymous wildtype variants were assessed for fitness score versus mean count. **d)** A minimum requirement of 10 read counts per variant was set for the drug-escape dataset. **e)** A minimum requirement of 20 read counts per variant was set for the drug-escape dataset. **f)** The average number of reads per variant from PacBio data was determined from the distribution of synonymous wildtype variant drug-escape fitness scores against the mean read count in PacBio sequencing data. Variants with less than 5.5 reads were discarded. Source data are provided as a Source Data file.

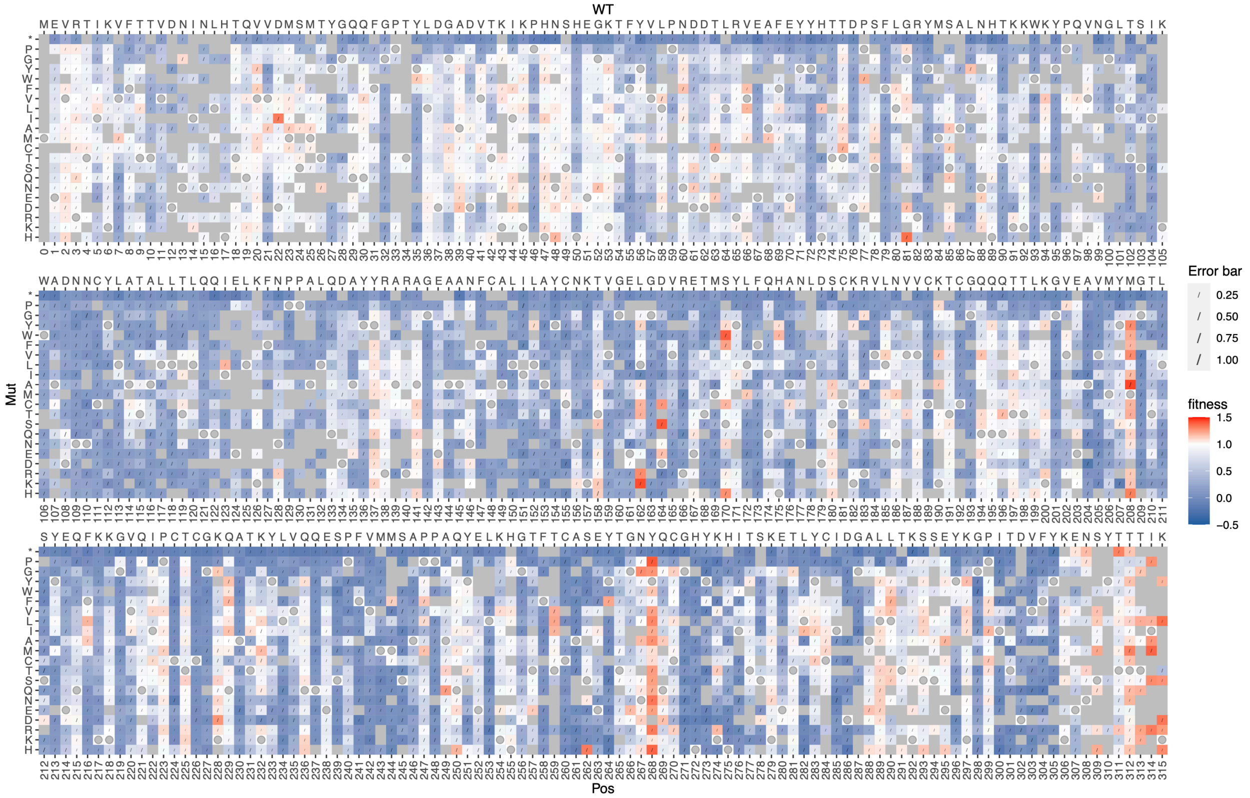

Supplementary Figure 13. **Sequence-function map of normalized combined drug-escape fitness scores after applying the filters outlined in Extended Data Fig. 12.** The bottom x-axis indicates the position; the top x-axis indicates WT sequence; and the y-axis indicates the mutation. Fitness is shown in a three-color gradient with red indicating escape variants, blue non-escape variants, and the white point set at the wild-type score of 1. Grey indicates missing variants. Wildtype variants are marked with a circle. Errors (sigma) are indicated with a line. Source data are provided as a Source Data file.

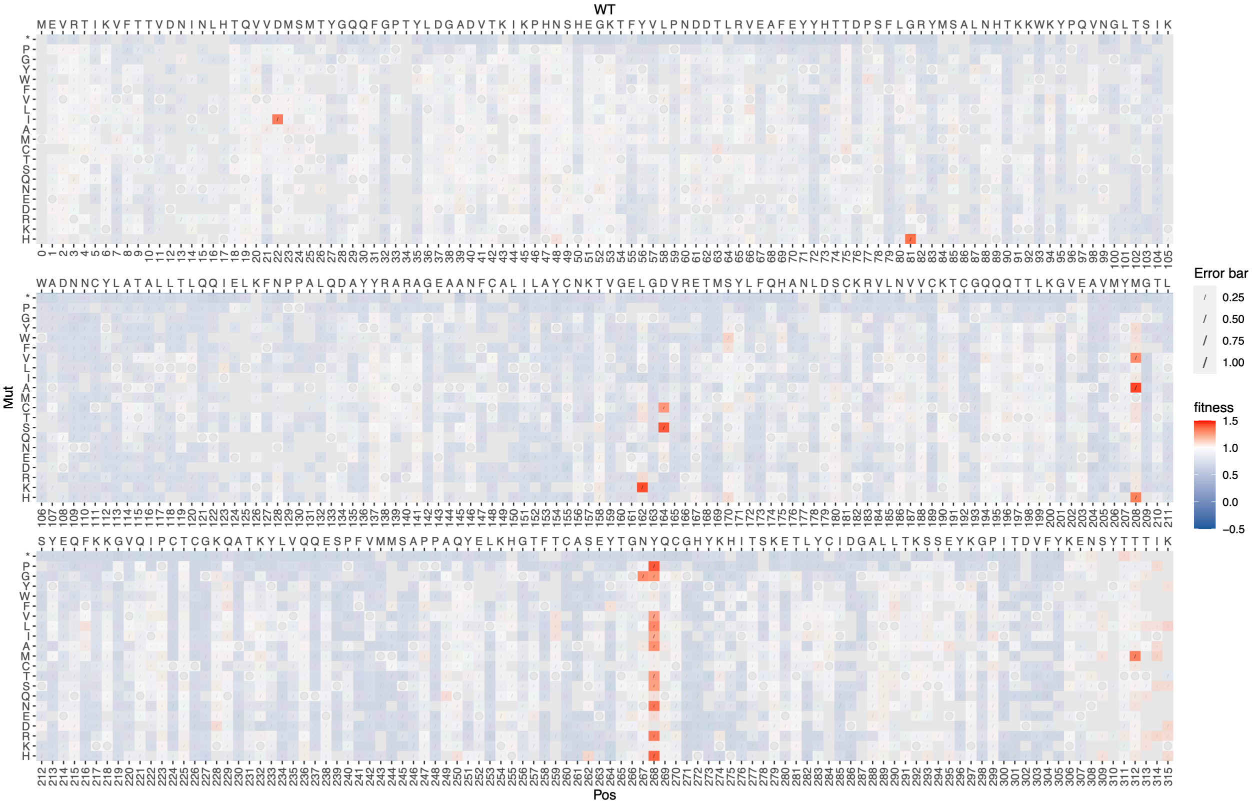

Supplementary Figure 14. **Drug-escape variants falling in the gate defined in Fig. 4c**. The sequence function map in Extended Data Fig. 13 was masked with a semi-transparent white layer except for variants identified as candidate escape variants, shown in shades of red. Source data are provided as a Source Data file.

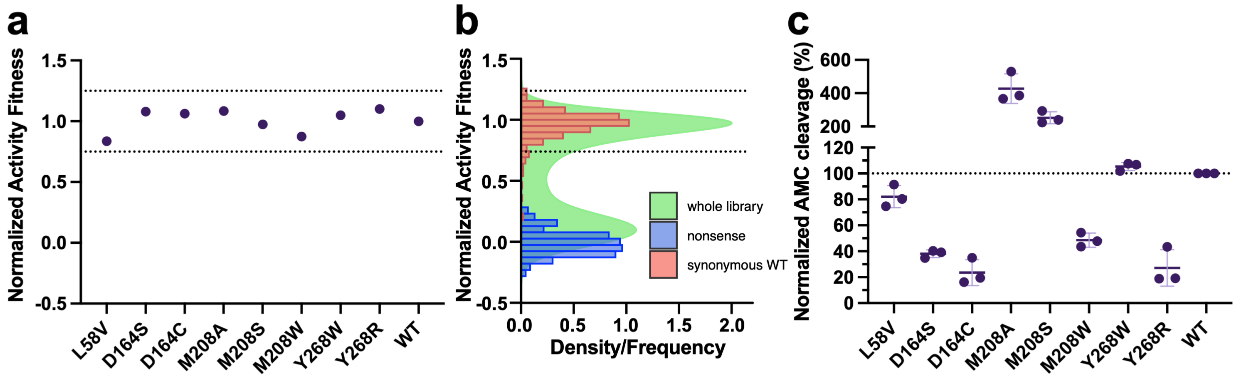

Supplementary Figure 15. **Comparison of DMS activity fitness scores (at steady state) with activity of recombinant proteins (initial rates). a**) Normalized activity fitness scores extracted from the DMS activity dataset. Dashed lines indicate wildtype-like activity determined from the graph in (b). **b)** The distribution of normalized PLpro activity fitness scores for the whole library (density; green), overlaid with the frequency of scores from synonymous wildtype variants (red; set at 1) and nonsense variants at positions 1-305 (blue; set at 0). **c)** Z-RLRGG-AMC cleavage by recombinant PLpro variants after 2 h incubation at room temperature. All data is normalized to wildtype (100%). Data is from three independent experiments. Error bars indicates mean ± SD. Source data are provided as a Source Data file.

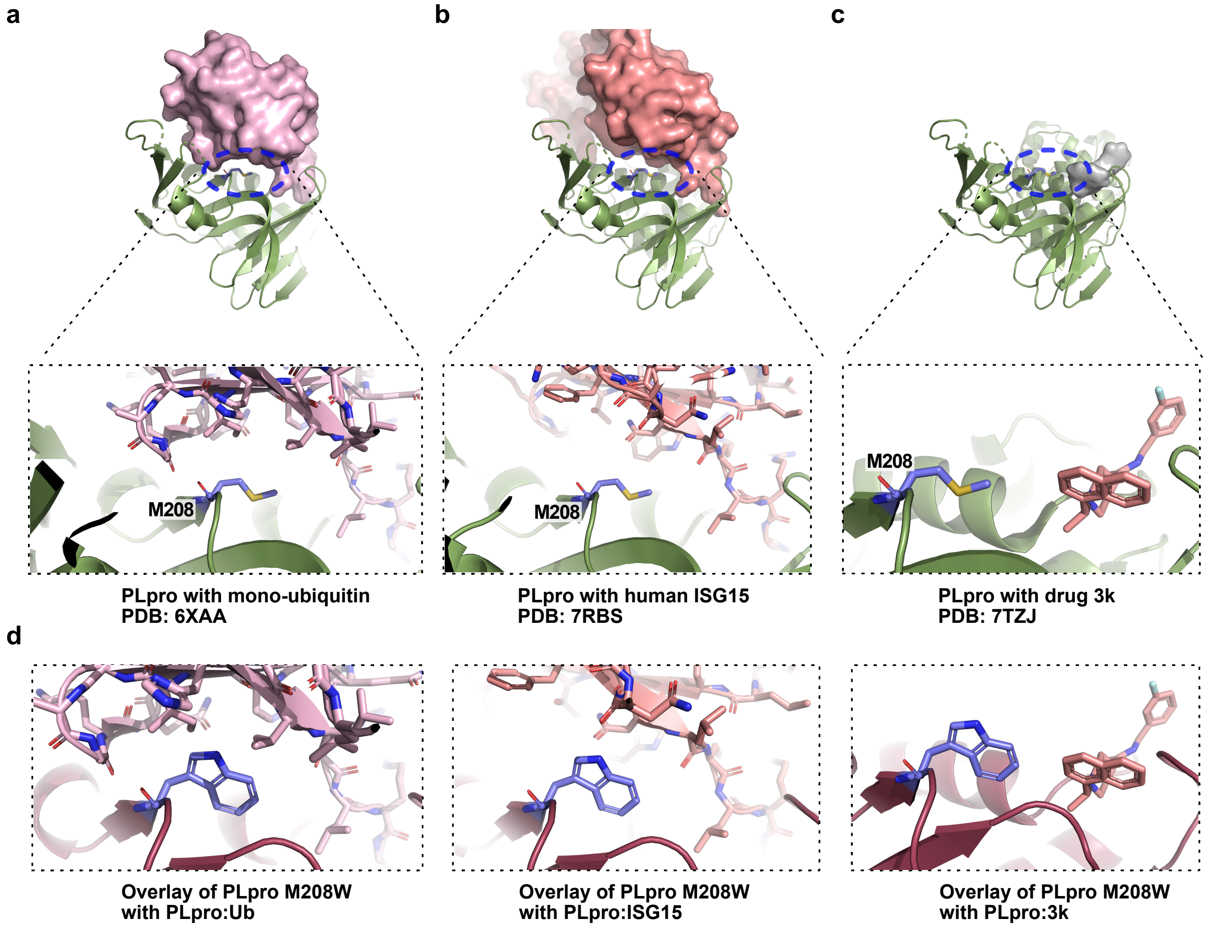

Supplementary Figure 16. **Met208 does not directly contact substrate or inhibitor. a)** PLpro (green) with Ub (pink) (PDB: 6XAA)[1] **b)** PLpro (green) with human ISG15 (red) (PDB: 7RBS)[2] **c)** PLpro (green) with 3k (grey) (PDB: 7TZJ)[3]. The location of Met208 is highlighted in the blue circle. Insets zoom in on Met208 and surrounding residues on substrates (in stick). **d)** Overlay of PLpro M208W with PLpro:Ub, PLpro:ISG15 and PLpro:3k.

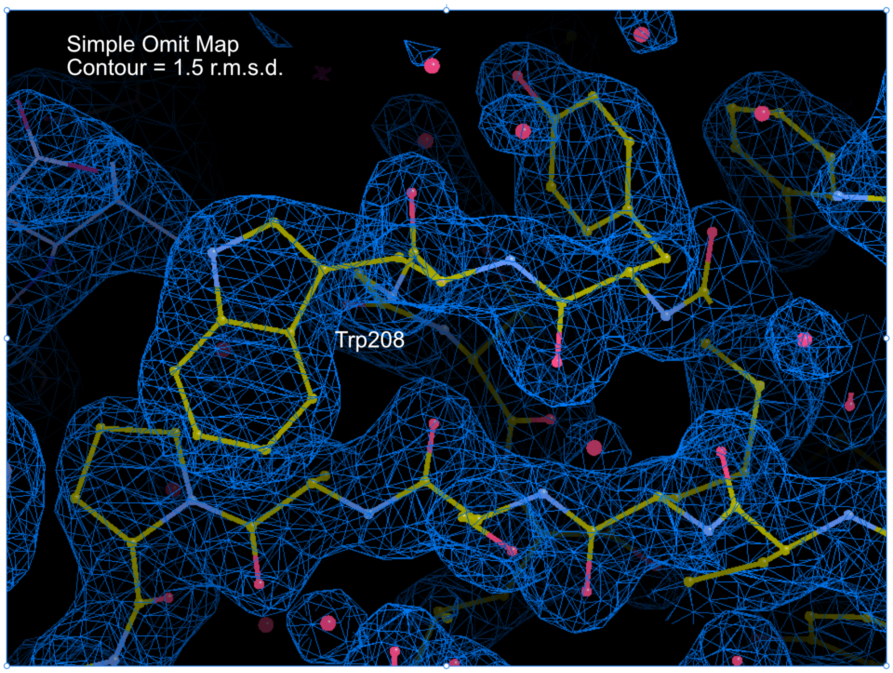

Supplementary Figure 17. **Omit map density around M208W mutation in 8VEC.** A simple omit map was calculated in Phenix and contoured to 1.5 rmsd.

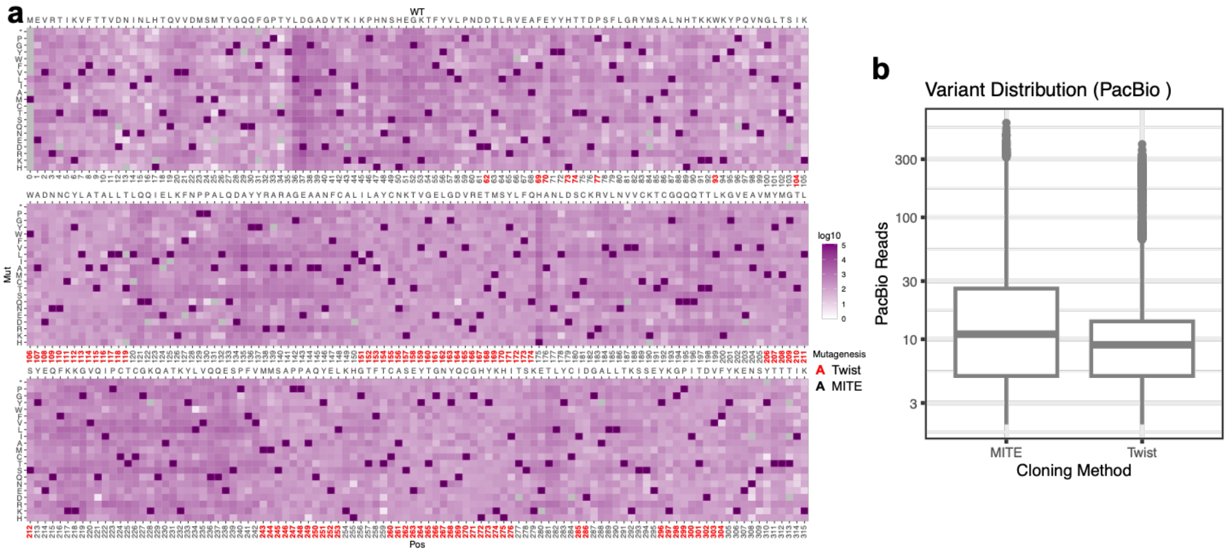

Supplementary Figure 18. **Counts map of PLpro PacBio Sequencing**. **a)** All PacBio reads from barcodes aligning to the same variants are summed and presented as the total counts in the heatmap. Position numbers where Twist dsDNA was used during cloning are colored in red. Others were made with MITE mutagenesis. **b)** Number of reads per barcode in PacBio for MITE mutagenesis versus positions cloned with Twist dsDNA. The center line of the BoxPlot represents the median value. The box's lower border marks the first quartile, and the upper border indicates the third quartile. The whiskers extend to 1.5 times interquartile range below/above the box. Outliers are displayed as dots outside the whiskers. Source data are provided as a Source Data file.

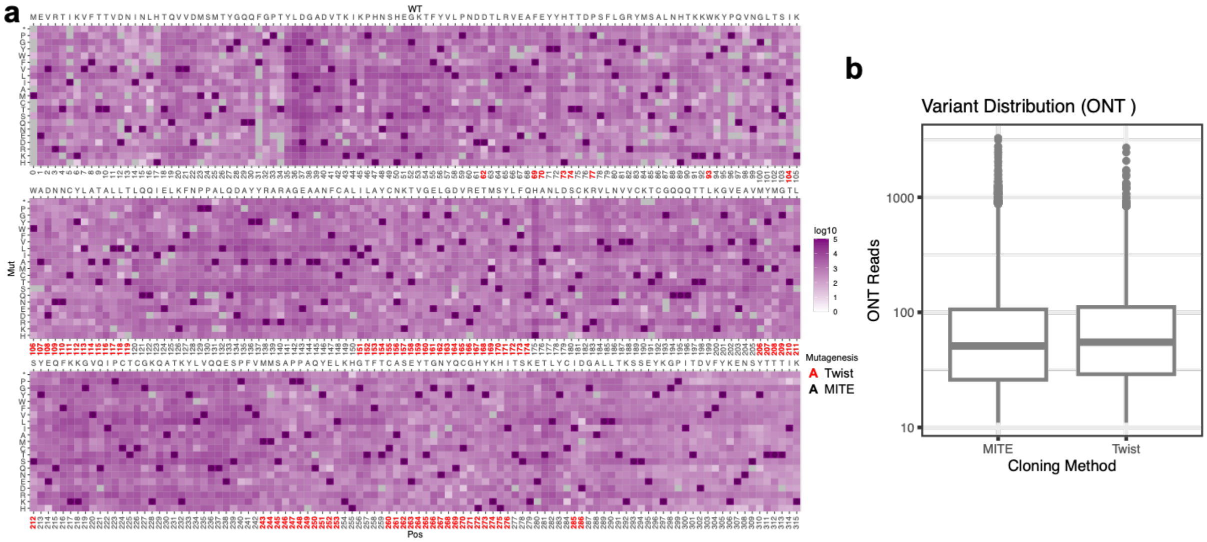

Supplementary Figure 19. **Counts map of PLpro-mClover3 Nanopore Sequencing**. **a)** All Nanopore reads from barcodes aligning to the same variants are summed and presented as the total counts in the heatmap. Position numbers where Twist dsDNA was used during cloning are colored in red. Others were made with MITE mutagenesis. **b)** Number of reads per barcode in PacBio for MITE mutagenesis versus positions cloned with Twist dsDNA. The center line of the BoxPlot represents the median value. The box's lower border marks the first quartile, and the upper border indicates the third quartile. The whiskers extend to 1.5 times interquartile range below/above the box. Outliers are displayed as dots outside the whiskers. Source data are provided as a Source Data file.

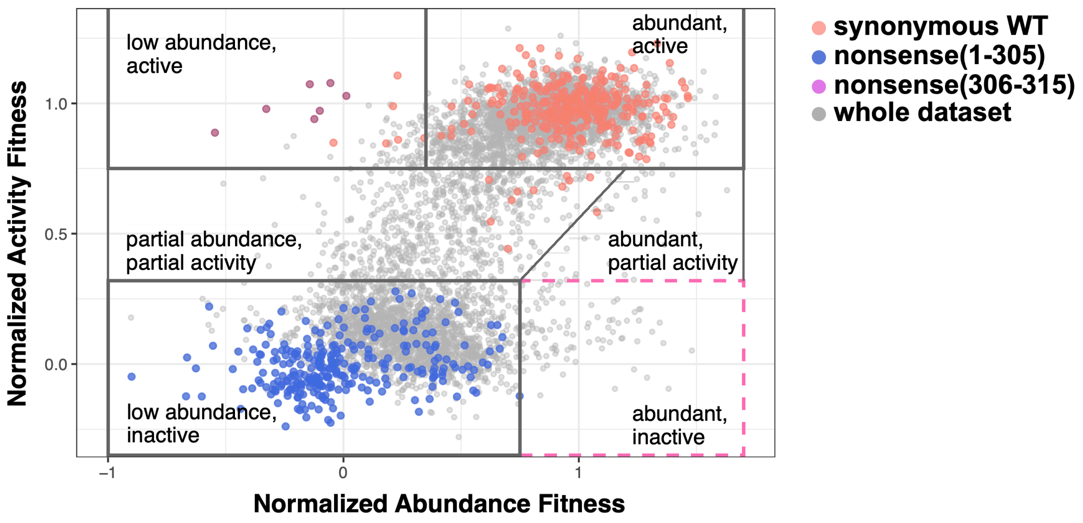

Supplementary Figure 20. **Gating strategy for classification of variants in Table 1.** The scatterplot shows the correlation between normalized abundance fitness scores (x-axis) and normalized activity fitness scores (y-axis). The library is depicted in grey, synonymous wildtype are shown in red and nonsense variants (positions 1-305) are shown in blue. Nonsense variants (positions 306-315), which are active, but do not make a GFP fusion protein are labelled in magenta. The gates are chosen based on the distribution of synonymous wildtype and nonsense variant datapoints. Source data are provided as a Source Data file.

	M208W (PDB: 8VEC)
**Wavelength**	0.9537 Å
**Resolution range**	47.5 - 2.002 (2.073 - 2.002)
**Space group**	P 21 21 2
**Unit cell**	57.958 85.47 82.88 90 90 90
**Total reflections**	210621 (20864)
**Unique reflections**	28423 (2601)
**Multiplicity**	7.4 (7.4)
**Completeness (%)**	99.19 (92.63)
**Mean I/sigma(I)**	13.08 (1.52)
**Wilson B-factor**	32.92
**R-merge**	0.1138 (1.305)
**R-meas**	0.1225 (1.406)
**R-pim**	0.0447 (0.516)
**CC1/2**	0.999 (0.495)
**CC***	1 (0.814)
**Reflections used in refinement**	28209 (2602)
**Reflections used for R-free**	2003 (180)
**R-work**	0.1738 (0.2616)
**R-free**	0.2170 (0.2932)
**CC(work)**	0.968 (0.750)
**CC(free)**	0.945 (0.718)
**Number of non-hydrogen atoms**	2750
**macromolecules**	2530
**ligands**	1
**solvent**	219
**Protein residues**	318
**RMS(bonds)**	0.008
**RMS(angles)**	0.86
**Ramachandran favored (%)**	97.15
**Ramachandran allowed (%)**	2.85
**Ramachandran outliers (%)**	0.00
**Rotamer outliers (%)**	0.36
**Clashscore**	3.40
**Average B-factor**	36.74
**macromolecules**	36.24
**ligands**	75.70
**solvent**	42.34

Supplementary Table 1. **Data collection and refinement statistics.** Statistics for the highest-resolution shell are shown in parentheses.

PDB	Resolution (Å)	scaling factor (k)	translating factor (b)	Wilson b factor	transformed Wilson b factor
8fwn [https://doi.org/10.2210/pdb8FWN/pdb]	1.50	0.904	12.525	26.06	36.08
6wrh [https://doi.org/10.2210/pdb6WRH/pdb][4]	1.60	0.817	10.337	28.53	33.65
7d6h [https://doi.org/10.2210/pdb7D6H/pdb][5]	1.60	0.726	12.985	30.39	35.06
7qcg [https://doi.org/10.2210/pdb7QCG/pdb][6]	1.75	0.288	20.220	45.3	33.26
7qci [https://doi.org/10.2210/pdb7QCI/pdb][6]	1.76	0.244	20.949	49.39	33.00
7qcm [https://doi.org/10.2210/pdb7QCM/pdb][6]	1.77	0.280	20.200	47.23	33.41
6wzu [https://doi.org/10.2210/pdb6WZU/pdb][4]	1.79	0.731	5.850	36.07	32.21
7sgu [https://doi.org/10.2210/pdb7SGU/pdb]	1.79	0.483	9.817	45.22	31.67
7qcj [https://doi.org/10.2210/pdb7QCJ/pdb][6]	1.84	0.283	19.937	47.37	33.36
7qch [https://doi.org/10.2210/pdb7QCH/pdb][6]	1.88	0.202	20.536	60.97	32.87
7rbr [https://doi.org/10.2210/pdb7RBR/pdb][7]	1.88	0.404	14.749	43.87	32.48
7d7k [https://doi.org/10.2210/pdb7D7K/pdb][8]	1.90	0.542	9.964	47.83	35.91
7ofs [https://doi.org/10.2210/pdb7OFS/pdb][9]	1.90	0.317	16.590	51.79	33.02
7ybg [https://doi.org/10.2210/pdb7YBG/pdb]	1.90	0.890	6.293	34.69	37.16
7qck [https://doi.org/10.2210/pdb7QCK/pdb][6]	1.92	0.289	18.762	49.1	32.94
7jn2 [https://doi.org/10.2210/pdb7JN2/pdb][4]	1.93	0.300	15.205	54.38	31.53
7jit [https://doi.org/10.2210/pdb7JIT/pdb][4]	1.95	0.290	18.741	45.53	31.96

7oft [https://doi.org/10.2210/pdb7OFT/pdb][9]	1.95	0.440	12.755	46.8	33.34
7sgw [https://doi.org/10.2210/pdb7SGW/pdb]	1.95	0.397	13.500	46.74	32.07
7d47 [https://doi.org/10.2210/pdb7D47/pdb]	1.97	0.173	24.400	40.95	31.49
7kok [https://doi.org/10.2210/pdb7KOK/pdb]	2.00	0.176	20.693	61.07	31.46
7sgv [https://doi.org/10.2210/pdb7SGV/pdb]	2.00	0.282	14.896	58.97	31.55
7sqe [https://doi.org/10.2210/pdb7SQE/pdb]	2.00	0.391	11.575	54.07	32.72
8vec (M208W) [https://doi.org/10.2210/pdb8VEC/pdb]	2.00	1.000	0.000	32.92	32.92
7koj [https://doi.org/10.2210/pdb7KOJ/pdb]	2.02	0.143	21.548	67.66	31.25
7m1y [https://doi.org/10.2210/pdb7M1Y/pdb]	2.02	0.477	5.331	56.59	32.35
7rzc [https://doi.org/10.2210/pdb7RZC/pdb]	2.04	0.372	14.198	48.75	32.32
7jiv [https://doi.org/10.2210/pdb7JIV/pdb]	2.05	0.184	19.930	61.08	31.18
7jir [https://doi.org/10.2210/pdb7JIR/pdb][4]	2.09	0.179	20.151	62.68	31.37
7d7l [https://doi.org/10.2210/pdb7D7L/pdb][8]	2.11	0.378	15.574	52.43	35.38
8g62 [https://doi.org/10.2210/pdb8G62/pdb]	2.17	0.337	14.169	55.62	32.92
7lbr [https://doi.org/10.2210/pdb7LBR/pdb][10]	2.20	0.469	11.552	43.63	32.03
7jiw [https://doi.org/10.2210/pdb7JIW/pdb][4]	2.30	0.140	19.229	82.63	30.77
7llf [https://doi.org/10.2210/pdb7LLF/pdb][10]	2.30	0.439	8.254	52.87	31.47
7e35 [https://doi.org/10.2210/pdb7E35/pdb][5]	2.40	0.065	28.116	72.89	32.86

6xg3 [https://doi.org/10.2210/pdb6XG3/pdb][4]	2.48	0.423	8.345	55.08	31.64
7jrn [https://doi.org/10.2210/pdb7JRN/pdb][11]	2.48	0.478	14.575	37.16	32.34
7cjd [https://doi.org/10.2210/pdb7CJD/pdb][12]	2.50	0.309	11.939	75.1	35.11
7kol [https://doi.org/10.2210/pdb7KOL/pdb]	2.58	0.199	12.402	99.78	32.21
7cmd [https://doi.org/10.2210/pdb7CMD/pdb][12]	2.59	0.301	18.071	54.8	34.57
7tzj [https://doi.org/10.2210/pdb7TZJ/pdb][3]	2.66	0.204	19.615	57.63	31.40
7nt4 [https://doi.org/10.2210/pdb7NT4/pdb][13]	2.68	0.055	27.795	42.66	30.15
6w9c [https://doi.org/10.2210/pdb6W9C/pdb]	2.70	0.178	20.814	56.13	30.81
6xaa [https://doi.org/10.2210/pdb6XAA/pdb][14]	2.70	0.155	22.154	58.05	31.17
7krx [https://doi.org/10.2210/pdb7KRX/pdb]	2.72	0.107	20.030	103.1	31.10
7sdr [https://doi.org/10.2210/pdb7SDR/pdb]	2.72	0.305	13.450	59.66	31.67
6wuu [https://doi.org/10.2210/pdb6WUU/pdb][15]	2.79	0.247	12.312	80.91	32.32
7lbs [https://doi.org/10.2210/pdb7LBS/pdb][10]	2.80	0.412	22.909	21.67	31.84
6xa9 [https://doi.org/10.2210/pdb6XA9/pdb][14]	2.90	0.170	17.847	102	35.18
7llz [https://doi.org/10.2210/pdb7LLZ/pdb][10]	2.90	0.369	19.434	35.05	32.37
7los [https://doi.org/10.2210/pdb7LOS/pdb][10]	2.90	0.358	12.920	50.48	30.99
7rbs [https://doi.org/10.2210/pdb7RBS/pdb][4]	2.98	0.505	-3.065	76.1	35.39
8eua [https://doi.org/10.2210/pdb8EUA/pdb][16]	3.10	0.217	12.717	90.65	32.36

6yva [https://doi.org/10.2210/pdb6YVA/pdb][17]	3.18	0.052	26.191	87.6	30.72
8cx9 [https://doi.org/10.2210/pdb8CX9/pdb][18]	3.50	0.260	17.826	63.1	34.26

Supplementary Table 2 **PLpro structures used for B-factor analysis.** Columns are as indicated. The last column contains the transformed Wilson B-factor after applying the transformation factors in columns three and four.

	Abundant			Partially Abun-dant	Low Abundance			
Position	**Inactive**	**Partially active**	**Active**	**Partially active**	**Inactive**	**Active**	**Missing Data**	**Classification**
W106	**C,E,G,K,L,M,R,T,V**	*A,F,I,N,P,Y*					**D,H** *Q,S*	Oxyanion hole[19]; similar position to Gln19 in papain[20]
C111	**A,D,E,G,R,S,T**			*P*	**F,H,I,K,L,M,N,Q,V,W,Y**			Active site
T115	**E**		A,C,N,V	*D,G*	**F,H,I,K,L,M,P,Q,R,W,Y**		S*	Second shell
L162	**D,I,N**	*H,Q,W*	A,C,F,G,K,R,T,Y	*E,P,S*			V,M	Substrate binding[21]
G163	**A**				**C,D,E,F,H,I,K,L,M,P,Q,S,T,V,W,Y**		**R,N**	Interact with substrate C-terminus[22]
D164	**A,H,M,T,V,W,Y**	*G,N*	C,E		**I,K,L,R**		S **F,P** Q*	Substrate binding[22, 23]
R166	**C,N,T,W,Y**	*F*		*A,K*	**D,E,G,I,L,M,P,S,V**		**Q,H**	Lines the S4 pocket. Unknown, may stabilise D164[24]
R183	**C,E,F,I,L,M,V,Y**		A,G,S	*Q*	**D,H,K,N,P,T,W**			Unknown
Y213	**D,E**		F,I,L,M,V,W		**A,C,G,H,K,N,P,Q,R,S,T**			Unknown
M243	**H,Q,W**			*C,I,L,V*	**A,D,E,F,G,K,N,P,R,S,T,Y**			Unknown
P248	**A,C,G**	*V*			**D,E,F,H,I,K,L,M,N,Q,R,S,T,W,Y**			Substrate binding[17, 25]
Y264	**C,I,K,L,M,Q,W**		F		**A,D,E,G,H,N,P,R,S,T**		**V**	Substrate binding[26]
G266	**K,L,M,N,R**	*C,H,P,Y*	E	*F,S*			*W,V,I,Q,T,D* A*	BL2 flexibility

Q269	**P**	*D*	A,C,E,F,G,H,I,K,L,N,R,S,T,V,W,Y				M*	Substrate binding
C270	**P**		A,E,F,G,H,I,L,M,Q,R,S,T,V,W,Y				K,N<br>*D*	Second shell[27]
G271	**A,E,K,R,W,Y**				**C,D,F,H,I,L,M,N,P,S,T,V**		**Q**	Substrate binding[1, 22] & blocking loop flexibility, second shell
H272	**R,W**				**A,C,D,E,F,G,I,K,L,M,N,P,Q,S,T,V,Y**			Active site
D286	**E,N**				**A,F,G,I,K,L,P,Q,S,T,V,W,Y**		**R,C,H,M**	Active site
G287	**N**				**A,C,D,F,H,I,L,M,P,Q,R,S,T,V,W,Y**		**K,E**	Second shell
D302	**C,N,T**				**A,E,F,G,H,I,K,L,M,Q,R,S,V,W,Y**		**P**	Lines the S4 pocket<br>Unknown

Supplementary Table 3 Classification of Functionally Important Residues. The classifications are performed based on Extended Data Fig. 20. In the table, inactive variants are in bold; partially active variants are in italics; active variants are neither bolded nor in italics. For variants missing data, activity information is provided with the same font treatment, except for variants missing both activity and abundance scores, which are marked with an asterisk.

Supplementary References

1. Klemm T*, et al.* Mechanism and inhibition of the papain-like protease, PLpro, of SARS-CoV-2. *The EMBO Journal* **39**, e106275 (2020).

2. Wydorski PM*, et al.* Dual domain recognition determines SARS-CoV-2 PLpro selectivity for human ISG15 and K48-linked di-ubiquitin. *Nature Communications* **14**, 2366 (2023).

3. Calleja DJ*, et al.* Insights Into Drug Repurposing, as Well as Specificity and Compound Properties of Piperidine-Based SARS-CoV-2 PLpro Inhibitors. *Front Chem* **10**, 861209 (2022).

4. Osipiuk J*, et al.* Structure of papain-like protease from SARS-CoV-2 and its complexes with non-covalent inhibitors. *Nature Communications* **12**, 743 (2021).

5. Shan H*, et al.* Development of potent and selective inhibitors targeting the papain-like protease of SARS-CoV-2. *Cell Chem Biol* **28**, 855-865.e859 (2021).

6. Ewert W*, et al.* Hydrazones and Thiosemicarbazones Targeting Protein-Protein-Interactions of SARS-CoV-2 Papain-like Protease. *Front Chem* **10**, 832431 (2022).

7. Wydorski PM*, et al.* Dual domain recognition determines SARS-CoV-2 PLpro selectivity for human ISG15 and K48-linked di-ubiquitin. *Nat Commun* **14**, 2366 (2023).

8. Zhao Y*, et al.* High-throughput screening identifies established drugs as SARS-CoV-2 PLpro inhibitors. *Protein Cell* **12**, 877-888 (2021).

9. Srinivasan V*, et al.* Antiviral activity of natural phenolic compounds in complex at an allosteric site of SARS-CoV-2 papain-like protease. *Commun Biol* **5**, 805 (2022).

10. Shen Z*, et al.* Potent, Novel SARS-CoV-2 PLpro Inhibitors Block Viral Replication in Monkey and Human Cell Cultures. *bioRxiv*, (2021).

11. Ma C*, et al.* Discovery of SARS-CoV-2 Papain-like Protease Inhibitors through a Combination of High-Throughput Screening and a FlipGFP-Based Reporter Assay. *ACS Cent Sci* **7**, 1245-1260 (2021).

12. Gao X*, et al.* Crystal structure of SARS-CoV-2 papain-like protease. *Acta Pharm Sin B* **11**, 237-245 (2021).

13. Napolitano V*, et al.* Acriflavine, a clinically approved drug, inhibits SARS-CoV-2 and other betacoronaviruses. *Cell Chem Biol* **29**, 774-784.e778 (2022).

14. Klemm T*, et al.* Mechanism and inhibition of the papain-like protease, PLpro, of SARS-CoV-2. *Embo j* **39**, e106275 (2020).

15. Rut W*, et al.* Activity profiling and crystal structures of inhibitor-bound SARS-CoV-2 papain-like protease: A framework for anti-COVID-19 drug design. *Sci Adv* **6**, (2020).

16. Sanders BC*, et al.* Potent and selective covalent inhibition of the papain-like protease from SARS-CoV-2. *Nat Commun* **14**, 1733 (2023).

17. Shin D, *et al.* Papain-like protease regulates SARS-CoV-2 viral spread and innate immunity. *Nature* **587**, 657-662 (2020).

18. van Vliet VJE, *et al.* Ubiquitin variants potently inhibit SARS-CoV-2 PLpro and viral replication via a novel site distal to the protease active site. *PLoS Pathog* **18**, e1011065 (2022).

19. Báez-Santos YM, St John SE, Mesecar AD. The SARS-coronavirus papain-like protease: structure, function and inhibition by designed antiviral compounds. *Antiviral Res* **115**, 21-38 (2015).

20. Ménard R, *et al.* Contribution of the glutamine 19 side chain to transition-state stabilization in the oxyanion hole of papain. *Biochemistry* **30**, 8924-8928 (1991).

21. Ratia K, Saikatendu KS, Santarsiero BD, Barretto N, Baker SC, Stevens RC, Mesecar AD. Severe acute respiratory syndrome coronavirus papain-like protease: Structure of a viral deubiquitinating enzyme. *Proceedings of the National Academy of Sciences* **103**, 5717-5722 (2006).

22. Fu Z, *et al.* The complex structure of GRL0617 and SARS-CoV-2 PLpro reveals a hot spot for antiviral drug discovery. *Nature Communications* **12**, 488 (2021).

23. Perlinska AP, *et al.* Amino acid variants of SARS-CoV-2 papain-like protease have impact on drug binding. *PLOS Computational Biology* **18**, e1010667 (2022).

24. Ma C, *et al.* Discovery of SARS-CoV-2 Papain-like Protease Inhibitors through a Combination of High-Throughput Screening and a FlipGFP-Based Reporter Assay. *ACS Central Science* **7**, 1245-1260 (2021).

25. Rut W, *et al.* Activity profiling and crystal structures of inhibitor-bound SARS-CoV-2 papain-like protease: A framework for anti–COVID-19 drug design. *Science Advances* **6**, eabd4596 (2020).

26. Zhao Y, *et al.* High-throughput screening identifies established drugs as SARS-CoV-2 PLpro inhibitors. *Protein & Cell* **12**, 877-888 (2021).

27. Shao Q, Xiong M, Li J, Hu H, Su H, Xu Y. Unraveling the catalytic mechanism of SARS-CoV-2 papain-like protease with allosteric modulation of C270 mutation using multiscale computational approaches. *Chemical Science* **14**, 4681-4696 (2023).
