# Supplementary material for: Mutational profiling of SARS-CoV-2 papain-like protease reveals requirements for function, structure, and drug escape
Source: Nat Commun. 2024 Jul 23;15:6219. doi: 10.1038/s41467-024-50566-9 (PMC11266423; doi:10.1038/s41467-024-50566-9)
Supplement: Supplementary file 3 — Description of Additional Supplementary Files [file 41467_2024_50566_MOESM3_ESM.pdf]

Description of Additional Supplementary Files

Title: Supplementary Data 1 - **Individual replicate activity fitness scores by variant**
Description: Each graph is headed by the wildtype residue and position. Variants are indicated on the *x*-axis. Scores are indicated on the *y*-axis, which is limited to –0.5-1.5 for clarity. Scores within the scale are solid circles. Scores falling above or below the scale are marked as x and placed at 1.5 or -0.5, respectively. Colors indicate variant type. Dark blue - basic, red - acidic, orange - polar, green - aliphatic, light blue - aromatic and black - Gly, Pro or Stop.

Title: Supplementary Data 2 - **List of circulating PLpro sequences**
Description: The excel file provides a list of PLpro circulating variants.

Title: Supplementary Data 3 - **Alignment of Coronaviridae PLpro sequences**
Description: The excel file provides the alignments between different Coronavirus PLpro sequences.

Title: Supplementary Data 4 - **Individual replicate abundance fitness scores by variant**
Description: Each graph is headed by the wildtype residue and position. Variants are indicated on the *x*-axis. Scores are indicated on the *y*-axis, which is limited to –0.5-1.5 for clarity. Scores within the scale are solid circles. Scores falling above or below the scale are marked as x and placed at 1.5 or -0.5, respectively. Colors indicate variant type. Dark blue - basic, red - acidic, orange - polar, green - aliphatic, light blue - aromatic and black - Gly, Pro or Stop.
