# Supplementary figures and images for: Mutational profiling of SARS-CoV-2 papain-like protease reveals requirements for function, structure, and drug escape
Source: Nat Commun. 2024 Jul 23;15:6219. doi: 10.1038/s41467-024-50566-9 (PMC11266423; doi:10.1038/s41467-024-50566-9)

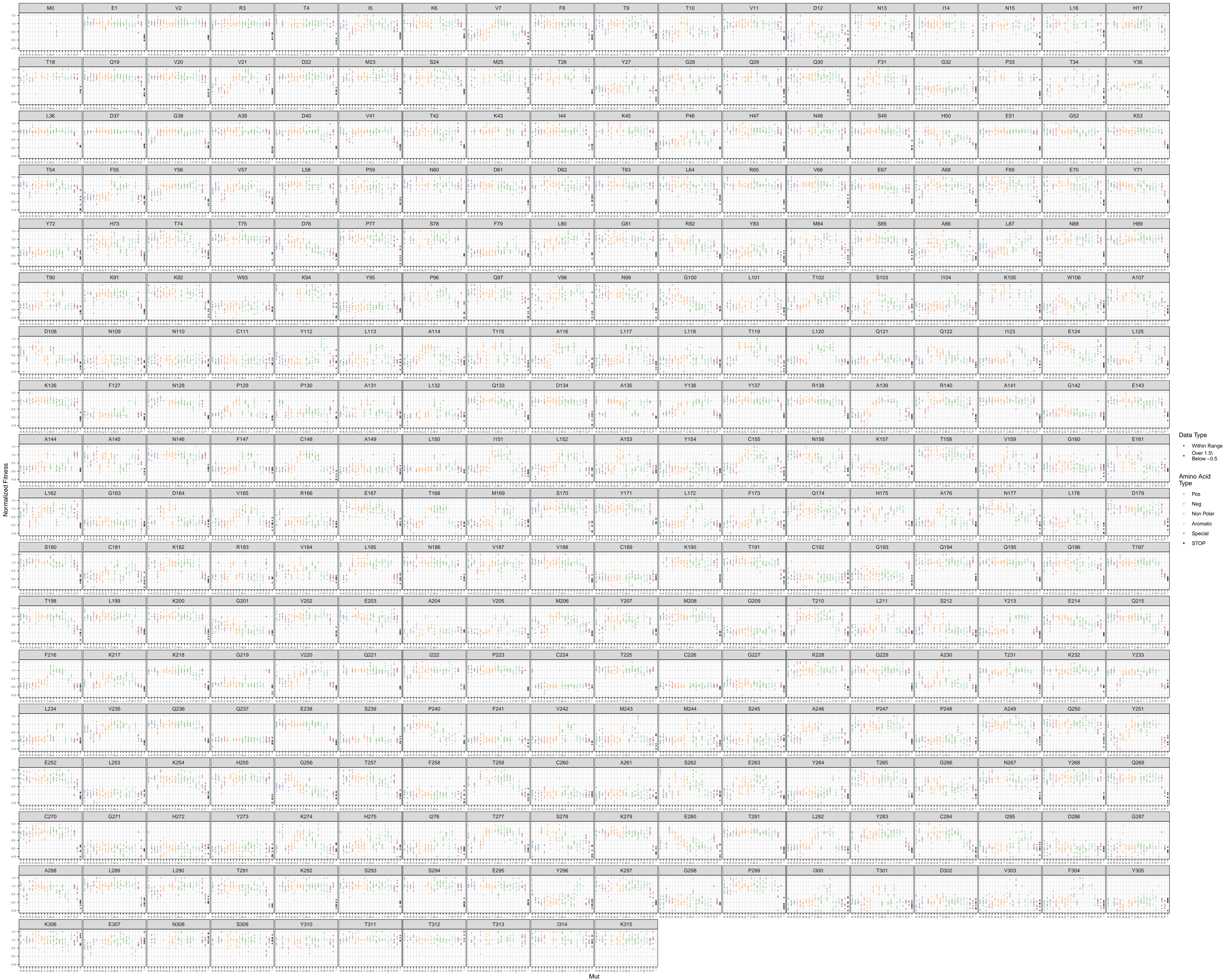

Supplement: Supplementary file 4 — Supplementary Data 1 [file 41467_2024_50566_MOESM4_ESM.pdf]
