# Supplementary material for: Mutational profiling of SARS-CoV-2 papain-like protease reveals requirements for function, structure, and drug escape
Source: Nat Commun. 2024 Jul 23;15:6219. doi: 10.1038/s41467-024-50566-9 (PMC11266423; doi:10.1038/s41467-024-50566-9)
Supplement: Supplementary file 8 — Reporting Summary [file 41467_2024_50566_MOESM8_ESM.pdf]

Corresponding author(s): Melissa J. Call

Last updated by author(s): Jul 1, 2024

Reporting Summary

Nature Portfolio wishes to improve the reproducibility of the work that we publish. This form provides structure for consistency and transparency in reporting. For further information on Nature Portfolio policies, see our Editorial Policies and the Editorial Policy Checklist.

Statistics

For all statistical analyses, confirm that the following items are present in the figure legend, table legend, main text, or Methods section.

n/a	Confirmed	
☐	☒	The exact sample size (*n*) for each experimental group/condition, given as a discrete number and unit of measurement
☐	☒	A statement on whether measurements were taken from distinct samples or whether the same sample was measured repeatedly
☒	☐	The statistical test(s) used AND whether they are one- or two-sided<br>*Only common tests should be described solely by name; describe more complex techniques in the Methods section.*
☒	☐	A description of all covariates tested
☐	☒	A description of any assumptions or corrections, such as tests of normality and adjustment for multiple comparisons
☐	☒	A full description of the statistical parameters including central tendency (e.g. means) or other basic estimates (e.g. regression coefficient) AND variation (e.g. standard deviation) or associated estimates of uncertainty (e.g. confidence intervals)
☒	☐	For null hypothesis testing, the test statistic (e.g. *F*, *t*, *r*) with confidence intervals, effect sizes, degrees of freedom and *P* value noted<br>*Give P values as exact values whenever suitable.*
☒	☐	For Bayesian analysis, information on the choice of priors and Markov chain Monte Carlo settings
☒	☐	For hierarchical and complex designs, identification of the appropriate level for tests and full reporting of outcomes
☒	☐	Estimates of effect sizes (e.g. Cohen's *d*, Pearson's *r*), indicating how they were calculated

Our web collection on statistics for biologists contains articles on many of the points above.

Software and code

Policy information about availability of computer code

Data collection	DNA sequencing data in this study was provided by WEHI Genomic hub and AGRF.<br>PLpro structure information was downloaded directly from Protein Data Bank (https://www.rcsb.org).<br>Thermal Shift Assay data was collected with Bio-Rad CFX Manager 3.0 (3.0 1224. 1015).<br>Flow Cytometry data was collected with BD FACSDiva Software v9.1.<br>CLARIOstar plate reader 5.70 R2 was used for measuring AMC/Rhodamine emission
Data analysis	Next-Generation Sequencing data processing: Cutadapt v3.4; UMI_tools v1.1.4; dorado v0.5.2; seqkit v2.61; minimap2 v2.17-r941; medaka v1.113; racon v1.5.0; R studio v4.2.0<br>Biochemical data: Prism 9<br>Flow Cytometry data: FlowJo 10.9.0<br>Crystallization data: XDS (Nov 1, 2016 BUILT=20161205); Phenix (1.10.1-4487), Truncate (version 8.0.000), Aimless (version 0.7.8), Phaser (2.8.3), Coot (0.9.8.92 EL ccp4)<br>Protein structures analysis: Pymol (version 2.5.7)<br>Code used in this study is available in Code Ocean (https://doi.org/10.24433/CO.0426220.v1)

For manuscripts utilizing custom algorithms or software that are central to the research but not yet described in published literature, software must be made available to editors and reviewers. We strongly encourage code deposition in a community repository (e.g. GitHub). See the Nature Portfolio guidelines for submitting code & software for further information.

Data

Policy information about availability of data

All manuscripts must include a data availability statement. This statement should provide the following information, where applicable:

- Accession codes, unique identifiers, or web links for publicly available datasets
- A description of any restrictions on data availability
- For clinical datasets or third party data, please ensure that the statement adheres to our policy

The DMS data generated in this study have been deposited in the MaveDB database under accession code mavedb:00000672 [https://www.mavedb.org/experiment-sets/urn:mavedb:00000672]. The crystallography data generated in this study have been deposited in the PDB under accession code 8VEC [https://doi.org/10.2210/pdb8VEC/pdb]. The data used to generate figures in this study are provided in the Source Data file. The PDB files used in this study are available in the PDB and are listed in Supp. Table 2.

Research involving human participants, their data, or biological material

Policy information about studies with human participants or human data. See also policy information about sex, gender (identity/presentation), and sexual orientation and race, ethnicity and racism.

Reporting on sex and gender	No human participants used in this study. Commercially-available cell human-derived cell lines are described below.
Reporting on race, ethnicity, or other socially relevant groupings	N/A
Population characteristics	N/A
Recruitment	N/A
Ethics oversight	N/A

Note that full information on the approval of the study protocol must also be provided in the manuscript.

Field-specific reporting

Please select the one below that is the best fit for your research. If you are not sure, read the appropriate sections before making your selection.

- [x] Life sciences
- [ ] Behavioural & social sciences
- [ ] Ecological, evolutionary & environmental sciences

For a reference copy of the document with all sections, see nature.com/documents/nr-reporting-summary-flat.pdf

Life sciences study design

All studies must disclose on these points even when the disclosure is negative.

Sample size	We did not perform statistical calculations to determine the sample size. Instead, we ensured that at least two replicates were performed for DMS datasets, which already contain high levels of internal replication. This is, a common practice in the field. Some screens, such as the activity screen, were performed more than twice as we included dox/no dox controls in multiple screens. Followup experiments were all performed at least twice to ensure consistency between results, except for structure determination, which was performed from one crystal.
Data exclusions	DMS data was filtered based on the mean count of the input library variants or barcodes, with low count variants/barcodes removed. Variants with poor agreement among barcodes were also removed. The criteria is reported in the manuscript and filters were applied equally to all data.
Replication	Exact replication number has been provided in legends of all figures.
Randomization	Randomisation was not performed as there are no samples/organisms/participants allocated to experimental groups in this study. All samples orginate from a single source of cells or protein.
Blinding	This study involves evaulating the mutational effects on PLpro in a pooled screen and followup biochemical experiments and structure determination. Blinding is not standard practice for any of these approaches and was not attempted.

Reporting for specific materials, systems and methods

We require information from authors about some types of materials, experimental systems and methods used in many studies. Here, indicate whether each material, system or method listed is relevant to your study. If you are not sure if a list item applies to your research, read the appropriate section before selecting a response.

Materials & experimental systems

n/a	Involved in the study
[x]	[ ] Antibodies
[ ]	[x] Eukaryotic cell lines
[x]	[ ] Palaeontology and archaeology
[x]	[ ] Animals and other organisms
[x]	[ ] Clinical data
[x]	[ ] Dual use research of concern
[x]	[ ] Plants

Methods

n/a	Involved in the study
[x]	[ ] ChIP-seq
[ ]	[x] Flow cytometry
[x]	[ ] MRI-based neuroimaging

Eukaryotic cell lines

Policy information about cell lines and Sex and Gender in Research

Cell line source(s)	We used authenticated HEK293T cell sourced from CellBank Australia, Cat# 12022001
Authentication	CellBank Australia supplies authenticated cell lines and morphology was consistent with the stated cell type.
Mycoplasma contamination	The parental cell line was tested for mycoplasma contamination by PCR and was negative.
Commonly misidentified lines (See ICLAC register)	No commonly misidentified cell lines were used in the study.

Plants

Seed stocks	N/A
Novel plant genotypes	N/A
Authentication	N/A

Flow Cytometry

Plots

Confirm that:

- [x] The axis labels state the marker and fluorochrome used (e.g. CD4-FITC).
- [x] The axis scales are clearly visible. Include numbers along axes only for bottom left plot of group (a 'group' is an analysis of identical markers).
- [x] All plots are contour plots with outliers or pseudocolor plots.
- [x] A numerical value for number of cells or percentage (with statistics) is provided.

Methodology

Sample preparation	HEK293T cells were lifted off via Trypsin digestion and passed through 100um cell-strainer, before proceeding to Flow Cytometry analysis or sorting.
Instrument	Flow Cytometry analysis was performed on BD FACSymphony A3 Cell Analyzer (BD Biosciences);<br>Flow Cytometry cell sorting was performed on BD FACsAria Fusion Flow Cytometer (BD Biosciences).
Software	BD FACSDiva software v9.1 was used for data collection;<br>FlowJo 10.9.0 was used for data analysis.
Cell population abundance	We sorted cells into FRET positive/negative pools (or mClover3 high/low pools for PLpro Abundance). The abundance was determined by the behaviours of variants transduced into cells. We mandated the amount of sorted cells to be over the number of originally transduced cells to maintain library diversity.
Gating strategy	FSC-A and SSC-A were used to gate live cells. mClover3 (B530/20) and FRET (B610/20) were used to select FRET positive/negative cells; For the Abundance assay, mClover3 was the criterial to select high abundance and low abundance cells. Gates

were dertermined so that the top half of the peak was used as high abundance and bottom half of the peak was used as low abundance. Details have been discussed in the figure legends and methods.

☒ Tick this box to confirm that a figure exemplifying the gating strategy is provided in the Supplementary Information.
