# Supplementary material for: Mutational profiling of SARS-CoV-2 papain-like protease reveals requirements for function, structure, and drug escape
Source: Nat Commun. 2024 Jul 23;15:6219. doi: 10.1038/s41467-024-50566-9 (PMC11266423; doi:10.1038/s41467-024-50566-9)
Supplement: Supplementary file 7 — Supplementary Data 4 [file 41467_2024_50566_MOESM7_ESM.pdf]

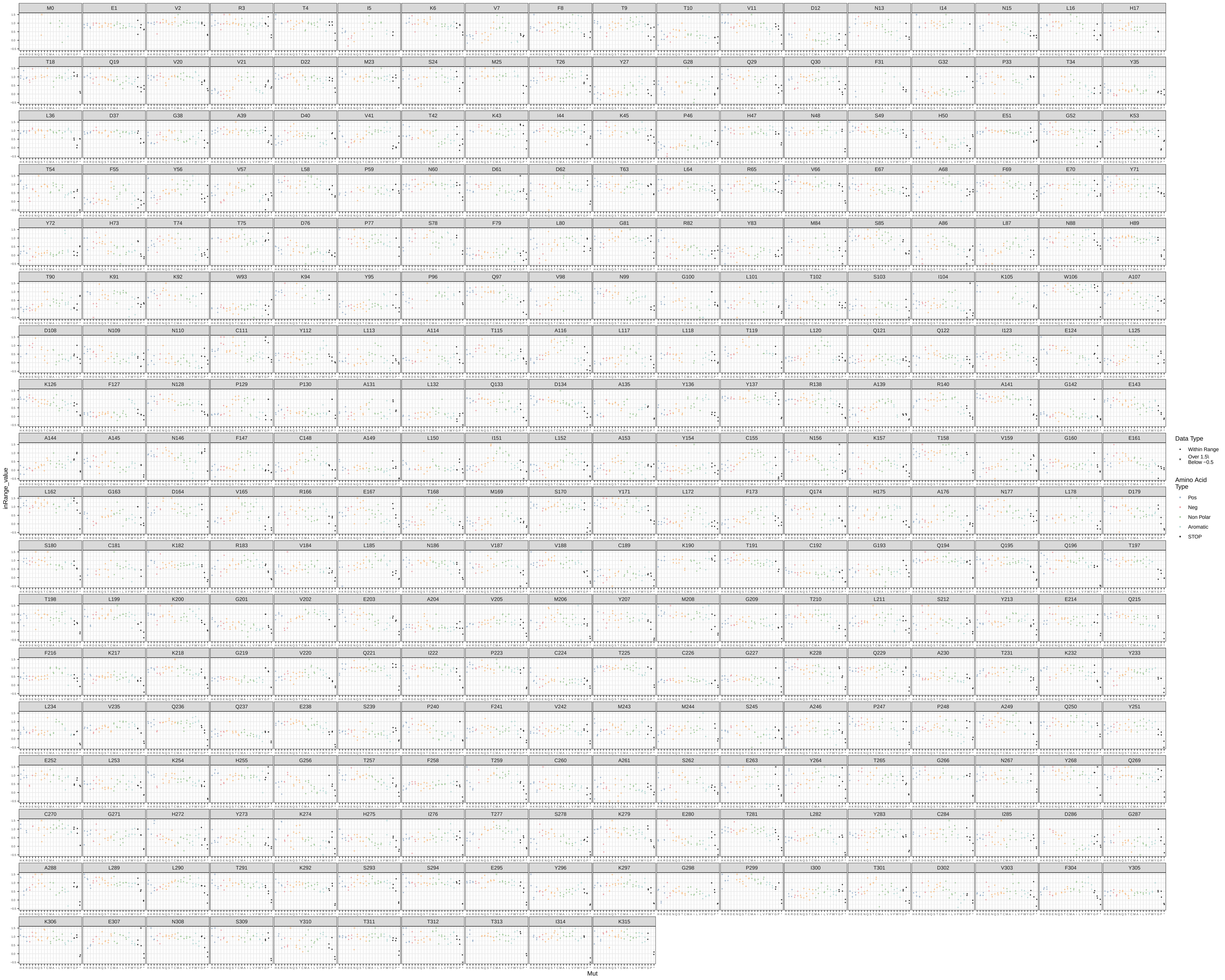
M0 E1 V2 R3 T4 I5 K6 V7 F8 T9 T10 V11 D12 N13 I14 N15 L16 H17
T18 Q19 V20 V21 D22 M23 S24 M25 T26 Y27 G28 Q29 Q30 F31 G32 P33 T34 Y35
L36 D37 G38 A39 D40 V41 T42 K43 I44 K45 P46 H47 N48 S49 H50 E51 G52 K53
T54 F55 Y56 V57 L58 P59 N60 D61 D62 T63 L64 R65 V66 E67 A68 F69 E70 Y71
Y72 H73 T74 T75 D76 P77 S78 F79 L80 G81 R82 Y83 M84 S85 A86 L87 N88 H89
T90 K91 K92 W93 K94 Y95 P96 Q97 V98 N99 G100 L101 T102 S103 I104 K105 W106 A107
D108 N109 N110 C111 Y112 L113 A114 T115 A116 L117 L118 T119 L120 Q121 Q122 I123 E124 L125
K126 F127 N128 P129 P130 A131 L132 Q133 D134 A135 Y136 Y137 R138 A139 R140 A141 G142 E143
A144 A145 N146 F147 C148 A149 L150 I151 L152 A153 Y154 C155 N156 K157 T158 V159 G160 E161
L162 G163 D164 V165 R166 E167 T168 M169 S170 Y171 L172 F173 Q174 H175 A176 N177 L178 D179
S180 C181 K182 R183 V184 L185 N186 V187 V188 C189 K190 T191 C192 G193 Q194 Q195 Q196 T197
T198 L199 K200 G201 V202 E203 A204 V205 M206 Y207 M208 G209 T210 L211 S212 Y213 E214 Q215
F216 K217 K218 G219 V220 Q221 I222 P223 C224 T225 C226 G227 K228 Q229 A230 T231 K232 Y233
L234 V235 Q236 Q237 E238 S239 P240 F241 V242 M243 M244 S245 A246 P247 P248 A249 Q250 Y251
E252 L253 K254 H255 G256 T257 F258 T259 C260 A261 S262 E263 Y264 T265 G266 N267 Y268 Q269
C270 G271 H272 Y273 K274 H275 I276 T277 S278 K279 E280 T281 L282 Y283 C284 I285 D286 G287
A288 L289 L290 T291 K292 S293 S294 E295 Y296 K297 G298 P299 I300 T301 D302 V303 F304 Y305
K306 E307 N308 S309 Y310 T311 T312 T313 I314 K315
1.5
1.0
0.5
0.0
-0.5
H K R D E N Q S T C M A I L V F W Y G P *
inRange_value
Mut
Data Type
Within Range
Over 1.5\
Below −0.5
Amino Acid Type
Pos
Neg
Non Polar
Aromatic
STOP
